# Molecular Epidemiology and Antibiotic Resistance Profiles of Methicillin-Resistant *Staphylococcus aureus* Strains in a Tertiary Hospital in China

**DOI:** 10.3389/fmicb.2017.00838

**Published:** 2017-05-12

**Authors:** Haishen Kong, Fei Yu, Weili Zhang, Xuefen Li, Hongxia Wang

**Affiliations:** ^1^State Key Laboratory for Diagnosis and Treatment of Infectious Diseases, Collaborative Innovation Center for Diagnosis and Treatment of Infectious Diseases, The First Affiliated Hospital, College of Medicine, Zhejiang UniversityHangzhou, China; ^2^Key Laboratory of Clinical In Vitro Diagnostic Techniques of Zhejiang Province, Department of Laboratory Medicine, The First Affiliated Hospital, College of Medicine, Zhejiang UniversityHangzhou, China; ^3^Department of Ultrasound, The First Affiliated Hospital, College of Medicine, Zhejiang UniversityHangzhou, China

**Keywords:** methicillin-resistant *Staphylococcus aureus*, *pvl*, genotyping, clonal complexes, multidrug resistance

## Abstract

Analysis of the genotypic characteristics and antimicrobial susceptibility patterns of methicillin-resistant *Staphylococcus aureus* (MRSA) is essential for the control and treatment of diseases caused by this important pathogen. In this study, MRSA isolates obtained from a tertiary caret hospital in China were subjected to *spa* typing, SCC*mec* typing, multiple locus sequence typing (MLST), and PCR targeting of the genes encoding Panton-Valentine leukocidin (PVL). The disk diffusion method was used to test the antimicrobial susceptibility of the isolates to 10 non-beta-lactam antibiotics. Among the 120 MRSA isolates studied, 18 *spa* types and 15 ST types were identified. The *spa* t311 type was the most common (a total of 60 isolates; 50%) among the study strains, and nearly all the t311 strains belonged to ST5, which is the most common ST type that was previously reported from China among the t002 isolates. ST5-II/t311 was the major prevalent clone (55, 45.8%), which was followed by ST5-II/t002 (12, 10.0%) and ST59-IV/t437 (11, 9.2%). PVL-encoding genes were found in 6.7% of the isolates. Although the ST5-II/t311 and ST5-II/t002 clones are different *spa* types, they shared the same resistance profile (clindamycin, erythromycin, and ciprofloxacin). Most isolates of the ST239-III/t037 clone were resistant to clindamycin, erythromycin, ciprofloxacin, gentamicin, tetracycline, and trimethoprim/sulfamethoxazole. By contrast, the MRSA isolates of the ST239-III/t030 clone were more resistant to rifampin, but they were susceptible to trimethoprim/sulfamethoxazole. Our data emphasize the need for ongoing epidemiologic surveillance.

## Introduction

*Staphylococcus aureus* (*S. aureus*) is a major pathogen that causes diseases ranging from wound infections to life-threatening bacteremia, ventilator-associated pneumonia, and sepsis. Methicillin-resistant *S. aureus* (MRSA) has emerged as a global pathogen in both hospital and community settings: multiple healthcare-associated MRSA (HA-MRSA) and community-associated MRSA (CA-MRSA) clones have disseminated internationally, which highlights the adaptation of the species to diverse ecological niches (Cuny et al., 2010). The distribution of MRSA clones is dynamic and geographically unique. Information regarding the molecular characteristics and antimicrobial susceptibility patterns of MRSA is essential for controlling and treating diseases caused by this medically important pathogen. As the epidemiology of infections with MRSA has changed, accurate information regarding the scope, and magnitude of MRSA infections is needed. The MRSA problem is not well-recognized in Chinese hospitals and communities. A nationwide, laboratory-based, multicenter surveillance study reported that ST239-III/t030, ST239-III/t037, and ST5-II/t002 were the predominant HA-MRSA clones, but the prevalence rates of these major clones varied markedly in different administrative regions (Xiao et al., 2013). A recent study (Cheng et al., 2013) also showed that these three major clones were associated with two characteristic resistance profiles: gentamicin/ciprofloxacin/rifampicin/levofloxacin for ST239-III/t030 clone and gentamicin/ciprofloxacin/clindamycin/erythromycin/tetracycline/levofloxacin/trimethoprim/sulfamethoxazole for ST239-III/t037 and ST5-II/t002 clones. Considering China's vast territory, the extent to which MRSA clones have spread into Chinese hospitals remains largely unknown. In this study, we determined the genotypes and resistance profiles of MRSA in a tertiary hospital in Eastern China that has 2,600 beds.

## Materials and methods

### MRSA isolates

This study was performed at the First Affiliated Hospital, College of Medicine, Zhejiang University, which is a tertiary hospital in China. A total of 120 non-duplicate clinical MRSA isolates obtained during January of each year from 2010 to 2014 were included in the current study. Identification of *S. aureus* was confirmed by MALDI-TOF MS (Bruker Daltonics, Bremen, Germany) and phenotypic methicillin resistance was assessed using the cefoxitin disk diffusion method in accordance with the Clinical and Laboratory Standard Institute guidelines (CLSI M100-S26) at our clinical laboratory, which has been accredited by the College of American Pathologists (Clinical Laboratory Standards Institute, 2016). The clinical sources of the isolates included the respiratory tract (*n* = 51), skin and soft tissue (*n* = 21), blood culture (*n* = 30), body fluid (*n* = 16), and urine (*n* = 2). A total of 85 (70.8%) isolates were obtained from inpatient cultures obtained >48 h after hospital admission, whereas 35 (29.2%) isolates were obtained from outpatients who had no history of hospitalization or invasive medical procedures in the prior year.

### DNA extraction

All isolates were cultured on blood agar and incubated overnight at 37°C. DNA was isolated using a QIAamp DNA Mini Kit (Qiagen, CA, USA) according to the manufacturer's instructions. The isolated DNA was used as the template for all PCR reactions.

### Confirmative identification of MRSA and detection of *pvl*

All *S. aureus* isolates with phenotypic methicillin resistance were first screened for the presence of the *mecA* gene, as previously described (Murakami et al., 1991). When *mecA*-negative isolates were found, further screening for the presence of the *mecC* gene was performed using a specific PCR method (Cuny et al., 2011). The isolates carrying the *mecA* or *mecC* gene were identified as MRSA (Ito et al., 2012). The presence of genes encoding Panton-Valentine leukocidin (PVL) was determined using a previously described PCR strategy (Larsen et al., 2008).

### Staphylococcal protein A (*spa*) typing

*Spa* typing involved PCR amplification and subsequent sequencing of the highly variable X region in the staphylococcal protein A using previously described primers *spa*-1113f and *spa*-1514r (Larsen et al., 2008). The *spa* sequence was analyzed using the *spa* database website (http://www.ridom.de/spaserver) to assign a unique *spa* type.

### SCC*mec* typing

SCC*mec* typing was determined using a multiplex polymerase chain reaction (PCR) strategy that was previously described as I–V (Boye et al., 2007). The MRSA isolates that had unanticipated fragments or that were lacking fragments by multiplex PCR were defined as non-typeable (NT).

### Multiple locus sequence typing (MLST)

MLST was performed on all isolates by sequencing the internal fragments of seven housekeeping genes (*arcC, aroE, glpF, gmK, pta, tpi*, and *yqiL*). The sequence profile and sequence type of each allele were determined according to the MLST database (http://saureus.mlst.net). The allelic profiles were assigned by comparing the sequences at each locus with those of the known alleles in the *S. aureus* MLST database and were accordingly defined as the sequence types (STs). For each locus, distinct allelic variants were assigned an allelic number and each unique combination of seven allele numbers was assigned a novel ST. Bionumerics v.6.06 (http://www.applied-maths.com) was used to generate a minimum spanning tree (MST) from the non-concatenated sequences of seven alleles of the 120 isolates.

### Antimicrobial susceptibility tests

Antimicrobial susceptibility testing of all the MRSA isolates to 10 non-beta-lactam antibiotics was determined using the disk diffusion method, which was performed according to the CLSI guidelines (M100-S26). The tested antimicrobial agents included erythromycin (ERY), clindamycin (CLI), rifampin (RIF), ciprofloxacin (CIP), gentamicin (GEN), tetracycline (TET), trimethoprim/sulfamethoxazole (SXT), linezolid (LZD), teicoplanin (TEC), and vancomycin (VAN) (Oxoid Ltd., Basingstoke, Hants, Chicago). *S. aureus* ATCC 25923 was used as a quality control strain.

### Definitions

The clone comprising 10% of the isolates was considered the major prevalent clone (Cheng et al., 2013). An isolate was considered multidrug-resistant (MDR) when the isolate was resistant to three or more classes of non-beta-lactam antimicrobial agents (Wang et al., 2008). Antibiotic resistance profiles (ARPs) of MRSA isolates were generated and included antimicrobial agents to which more than 70% of the MRSA isolates were resistant (Cheng et al., 2013).

### Ethics statement

This study was performed in accordance with the recommendations of The First Affiliated Hospital, College of Medicine, Zhejiang University and the Declaration of Helsinki. Written informed consent was obtained from all participating subjects.

## Results

### Genotyping and *pvl* detection

**Figure 1:**
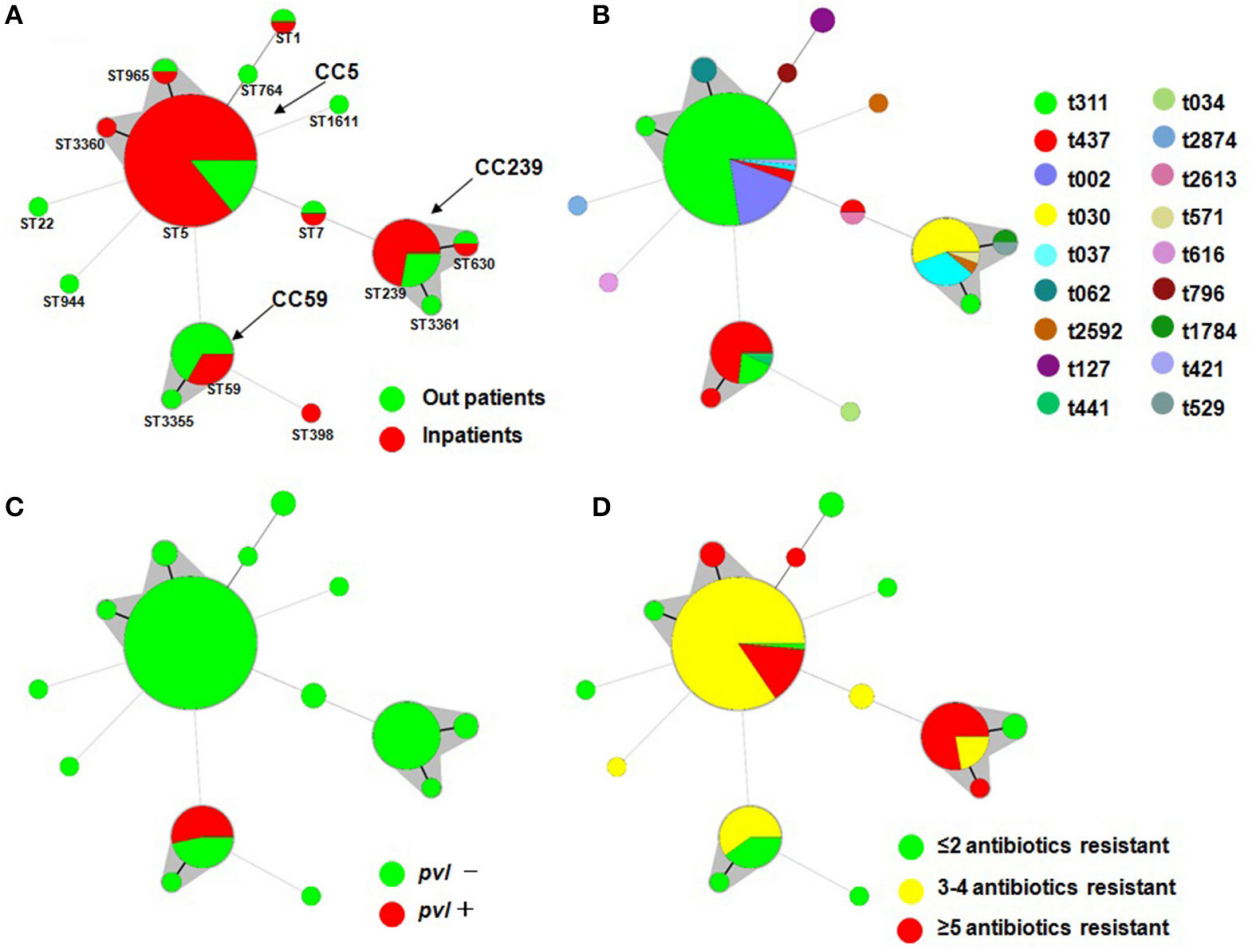
**Minimal Spanning Tree (MST) analysis of MRSA strains based on MLST data**. Each circle corresponds to an ST. The area of each circle corresponds to the number of isolates. The relationships between the strains are indicated by the connections between the isolates and the lengths of the branches linking them. Black lines connecting pairs of STs indicate that they differ in one allele (thick lines), two alleles (thin lines), or three to seven alleles (dashed lines, refer to ST22-ST5, ST944-ST5, ST59-ST5, ST7-ST5, ST1611-ST5, ST1-ST764, ST398-ST59 and ST7-ST239). Gray zones surround STs belonging to the same clonal complex (the clonal complexes were defined from this collection, and CC5 was predominant). Four MST graphs were separately generated based on the following associations: **(A)** ST vs. patients; **(B)** ST vs. *spa*; **(C)** ST vs. *pvl*; and **(D)** ST vs. multidrug resistance.

Among the 120 MRSA isolates studied, 15 different STs were identified. The three major STs were ST5 (71, 59.2%), ST239 (18, 15.0%), and ST59 (15, 12.5%). The remaining STs were ST1, ST7, ST22, ST398, ST630, ST764, ST944, ST965, ST1611, ST3355, ST3360, and ST3361, which had frequencies ranging from 0.8 to 1.7% (Figure 1). Among them, ST1611, ST3355, ST3360, and ST3361 were novel and have been deposited in the MLST database. SCC*mec* typing revealed that SCC*mec* II (75, 62.5%) was the most common, which was followed by SCC*mec* III (17, 14.2%), IV (16, 13.3%), and V (5, 4.2%). A total of 7 (5.8%) isolates were defined as NT. Further molecular characterizations showed that most isolates of ST5 carried SCC*mec* II, whereas ST239 carried SCC*mec* III and ST59 carried SCC*mec* IV. In addition, a total of 18 genotypes were identified in the 120 strains by *spa* typing. The most common *spa* types were t311 (60, 50.0%), t437 (15, 12.5%), t002 (12, 10.0%), t030 (10, 8.3%), and t037 (7, 5.8%). t311 was a common genotype that had been identified annually in this hospital at a prevalence ranging from 37.0 to 70.0%. The remaining 13 *spa* types accounted for a total of 13.4% of the isolates, none of which accounted for more than 2%. Five major *spa* types, including t311 (91.7% belonging to ST5), t437 (73.3%, ST59), t002 (100%, ST5), t030 (100%, ST239), and t037 (85.7%, ST239), prevailed in many departments at this hospital (data not shown). The most common genotype was ST5-II/t311 (55, 45.8%), which was followed by ST5-II/t002 (12, 10.0%), ST59-IV/t437 (11, 9.2%), ST239-III/t030 (10, 8.3%), and ST239-III/t037 (6, 5.0%).

Overall, *pvl* was detected in 8 MRSA isolates (6.7%) that were distributed annually, except for 2014 (Table 1). All *pvl-*positive isolates belonged to ST59-t437 (Figure 1).

**Table 1:** **Epidemiology of 120 MRSA isolates over 5 years including the distributions of SCC***mec, spa***, and MLST types**.

**Parameter**	**Year**					
	**2010**	**2011**	**2012**	**2013**	**2014**	**Total**
**No. of isolates identified**	**20**	**25**	**27**	**28**	**20**	**120**
ST-SCC*mec*/*spa* Type^*^						
ST5-II/t311	8 (40.0%)	8 (32.0%)	9 (33.3%)	16 (57.1%)	14 (70.0%)	55 (45.8%)
ST5-II/t002	3 (15.0%)	6 (24.0%)	1 (3.7%)	2 (7.1%)		12 (10.0%)
ST59-IV/t437	1 (5.0%)	3 (12.0%)	3 (11.1%)	3 (10.7%)	1 (5.0%)	11 (9.2%)
ST239-III/t030	5 (25.0%)	3 (12.0%)	1 (3.7%)		1 (5.0%)	10 (8.3%)
ST239-III/t037	3 (15.0%)	1 (4.0%)	2 (7.4%)			6 (5.0%)
ST59-IV/t311			1 (3.7%)	2 (7.1%)		3 (2.5%)
ST5-II/t437				2 (7.1%)		2 (1.7%)
ST5-III/t037		1 (4.0%)				1 (0.8%)
ST5-II/t421		1 (4.0%)				1 (0.8%)
ST59-NT/t441			1 (3.7%)			1 (0.8%)
ST239-NT/t2592			1 (3.7%)			1 (0.8%)
ST239-II/t571					1 (5.0%)	1 (0.8%)
ST1-NT/t127			1 (3.7%)			1 (0.8%)
ST1-IV/t127		1 (4.0%)				1 (0.8%)
ST7-NT/t2613			1 (3.7%)			1 (0.8%)
ST7-V/t437				1 (3.6%)		1 (0.8%)
ST22-V/t2874					1 (5.0%)	1 (0.8%)
ST398-II/t034				1 (3.6%)		1 (0.8%)
ST630-NT/t529				1 (3.6%)		1 (0.8%)
ST630-V/t1784					1 (5.0%)	1 (0.8%)
ST764-IV/t796					1 (5.0%)	1 (0.8%)
ST944-V/t616			1 (3.7%)			1 (0.8%)
ST965-NT/t062			1 (3.7%)			1 (0.8%)
ST965-II/t062			1 (3.7%)			1 (0.8%)
ST1611-NT/t2592			1 (3.7%)			1 (0.8%)
ST3355-V/t437			1 (3.7%)			1 (0.8%)
ST3360-II/t311			1 (3.7%)			1 (0.8%)
ST3361-II/t311		1 (4.0%)				1 (0.8%)
PVL-positive	1 (5%)	2 (8%)	4 (14.8%)	1 (3.6%)		8 (6.7%)

* *No. (%) of each molecular type in the year*.

### Antimicrobial resistance

All the MRSA isolates were susceptible to VAN, LZD, and TEC. The detailed antibiograms of the isolates for the other 7 non-beta-lactam antibiotics stratified by the MLST, SCC*mec*, and *spa* types are shown in Table 2. Isolates of the different genetic lineages exhibited distinct patterns of antibiotic susceptibilities. All the ST239 strains were MDR, with resistance to a median of 5 antibiotics for the t030 isolates (range, 4–7) and with a median of 6 antibiotics for the t037 isolates. Interestingly, ST5-II/t311 and ST5-II/t002 isolates shared the same ARPs, and they were frequently resistant to CLI (94.5–100%), ERY (96.7–100%), and CIP (98.2–100%), whereas ST239 isolates were generally resistant to CIP (100%), GEN (90–100%), and TET (100%). The t030 isolates were less likely than the t037 isolates to be resistant to CLI (60 and 100%, respectively) and ERY (60 and 100%, respectively). Notably, ST239-III/t030 isolates showed 100% resistance to RIF and 100% susceptibility to SXT, whereas ST239-III/t037 isolates showed the opposite pattern (Table 2).

**Table 2:** **Antibiotic resistant rates of 120 methicillin-resistant ***Staphylococcus aureus*** isolates by MLST, SCC***mec***, and ***spa*** types^**Δ**^**.

**Clone**	**% of isolates resistant to**							**ARPs**
	**CLI**	**ERY**	**CIP**	**GEN**	**TET**	**RIF**	**SXT**	
ST5-II/t311	94.5	96.7	98.2	20	45.5	3.6	3.6	CLI, ERY, CIP
ST5-II/t002	100	100	100	33.3	8.3	0	0	CLI, ERY, CIP
ST59-IV/t437	100	100	18.2	9.1	54.5	0	0	CLI, ERY
ST239-III/t030	60	60	100	90	100	100	0	CIP, GEN, TET, RIF
ST239-III/t037	100	100	100	100	100	0	100	CLI, ERY, CIP, GEN, TET, SXT

*CLI, clindamycin; ERY, erythromycin; CIP, ciprofloxacin; GEN, gentamicin; TET, tetracycline; RIF, rifampin; SXT, trimethoprim/sulfamethoxazole; ARPs, antimicrobial resistance profiles*.

Δ *Antibiotic-resistant strains included strains that tested as intermediate and resistant by the disk diffusion method. All 120 isolates were susceptible to vancomycin, teicoplanin, and linezolid*.

### MLST analysis and phylogenetic relationship

To determine the clonal relationship between the isolates, we used a Minimal Spanning Tree (MST) algorithm based on the allelic profiles. The 120 sequence-typed isolates were distributed into 9 isolates that did not belong to any clonal complexes (CCs) and 3 groups with a total of 111 isolates corresponding to CCs found in the database (Figure 1). The main detected CC was CC5, which consisted of three STs (ST5, ST965, and ST3360; ST5 was the primary founder). The second most frequently encountered CC, CC239, consisted of three STs (ST239, ST630, and ST3361; ST239 was the primary founder). The third encountered CC, CC59, consisted of two STs (ST59 and ST3355; ST59 was the primary founder). Another group was a doublet in which ST1 and ST764 were linked, and ST7, ST22, ST398, ST944, and ST1611 formed a simple complex (Figure 1).

The MST analysis revealed several interesting relationships with respect to *spa* type, patients, MDR phenotype and presence of the virulence gene (Figure 1). Three main STs were shared by more than 3 *spa* types (ST5 by 5, ST239 by 4, and ST59 by 3), and most ST5 and ST239 isolates were MDR and did not carry the *pvl* gene. It is noteworthy that ST59 consisted of 15 strains, 11 of which were t437, and all 8 *pvl*-positive strains belonged to t437. A total of 72.2% of ST239 isolates were resistant to more than five antibiotics, and only 14.1% of ST5 isolates were resistant to more than five antibiotics. In addition, 66.7% of the ST59 strains were isolated from outpatients, and 33.3% of ST59, 85.9% of ST5, and 72.2% of ST239 were isolated from inpatients.

## Discussion

MRSA is considered the most significant multidrug-resistant organism that causes infections, and the global spread of MRSA has been a major problem worldwide (Song et al., 2011; Klein et al., 2013). Analysis of the genotypic characteristics of MRSA clones is valuable for understanding the evolution and dissemination of MRSA (Stefani et al., 2012). In the present study, we characterized a cluster of MRSA strains obtained from a tertiary hospital in Hangzhou, China. There were limitations to this study. One major bias is the limited number of isolates analyzed. Nevertheless, the high prevalence of ST5-II/t311, a clone that was not previously popular in China or any other Asian country, is significant and striking.

In this study, we found that CC5 was the most common CC, which was followed by CC239 and CC59. This result does not agree with previous reports showing that the predominant CCs were CC239, CC5, and CC59 in China (Cheng et al., 2013; Xiao et al., 2013). Furthermore, ST5 (CC5) was the most predominant ST for SCC*mec* II isolates, and the most common *spa* type in the ST5 isolates was t311 (77.5%, 55/71). ST5 is the most common previously reported ST type from China among the t002 isolates (Xiao et al., 2013; Cheng et al., 2013). The repetitive sequence of t311 (26-23-17-34-20-17-12-17-16) is closely related to that of t002 (26-23-17-34-17-20-17-12-17-16), with only one repetition of difference between these two *spa* types. ST5-II was initially described as the main clone in the USA (Stefani et al., 2012) and Japan (Aires et al., 2000), and it was subsequently detected in several European and Asian countries (Baba et al., 2002; Song et al., 2011). ST5-II was prevalent in 2009 the Chinese cities of Shenyang and Dalian (Liu et al., 2009). ST5-II/t311 has been found in many other countries, such as Brazil, Nigerian, Angola, Argentina, and Oman (Camargo et al., 2013; Kolawole et al., 2013; Conceição et al., 2014; Egea et al., 2014; Udo et al., 2014). From 2010 to 2014, ST5-II/t311 has surpassed ST5-II/t002 as the predominant clone in our hospital; however, its mechanism remains to be further elucidated.

Of note, 15 (12.5%) of the ST59 isolates were identified in our MRSA collection, and more than half of these were PVL-positive. In addition, the ST59 isolates had less antibiotic resistance than ST239 and ST5 isolates. Clones harboring SCC*mec* IV or V and carrying PVL are usually reported in the context of community-onset infections, and ST59 is known to be the most common CA-MRSA clone among Chinese children (Geng et al., 2010; Wang et al., 2012). In Hong Kong and Taiwan, CA-MRSA clones, including ST59, have spread to hospital settings (Song et al., 2011). Dispersal is also facilitated by the emergence and persistence of MDR clones in hospitals. Our findings further suggest that hospitals in Mainland China are facing the same situation. The findings from this study further support that CA-MRSA strains usually remain susceptible to non-beta-lactam antibiotics and often carry the *pvl* gene that encodes PVL, a phage-borne leukotoxin (Okuma et al., 2002).

The association of MRSA ARPs with their molecular characteristics can provide useful information for the clinical selection of antibiotics. MRSA isolates also harbor numerous determinants associated with antibiotic resistance (Wendlandt et al., 2013; Yuan et al., 2013). The important finding from this study is that the five major clones were associated with four characteristic ARPs (Table 2). Despite having different *spa* types, ST5-II/t311 and ST5-II/t002 clones shared the same resistance profile (CLI, ERY, and CIP). Most isolates of ST239-III/t037 clones were resistant to CLI, ERY, CIP, GEN, TET, and SXT. By contrast, the MRSA isolates of the ST239-III/t030 clone were more resistant to RIF, but they were susceptible to SXT, which is concordant with previous reports (Chen et al., 2010; Cheng et al., 2013). This finding suggests that the clinical selection of antibiotics based on typing information is advantageous for treating patients with MRSA infections.

The present study revealed that ST5 was strongly associated with t311 type strains, which has gradually increased in prevalence during the past 5 years. The majority of ST5 strains were resistant to 3 or 4 non-beta-lactam antibiotics and non-ST5 strains harbored the *pvl* virulence gene. Ultimately, our dataset and analysis demonstrate that ST5-II/t311 is a major prevalent clone that is widespread in the hospital.

## Author contributions

HK and HW designed the experiments, HK and WZ conducted the experiment(s), and XL and FY analyzed the results. All authors reviewed the manuscript.

### Conflict of interest statement

The authors declare that the research was conducted in the absence of any commercial or financial relationships that could be construed as a potential conflict of interest.
